# Rapamycin and its analogues (rapalogs) for Tuberous Sclerosis Complex-associated tumors: a systematic review on non-randomized studies using meta-analysis

**DOI:** 10.1186/s13023-015-0317-7

**Published:** 2015-08-12

**Authors:** Teguh Haryo Sasongko, Nur Farrah Dila Ismail, Nik Mohamad Ariff Nik Abdul Malik, Z. A. M. H. Zabidi-Hussin

**Affiliations:** Human Genome Center, School of Medical Sciences, Universiti Sains Malaysia, USM Health Campus, 16150 Kubang Kerian, Kelantan Malaysia; Department of Pediatrics, School of Medical Sciences, Universiti Sains Malaysia, USM Health Campus, 16150 Kubang Kerian, Kelantan Malaysia; Center for Neuroscience Services and Research, Universiti Sains Malaysia, USM Health Campus, 16150 Kubang Kerian, Kelantan Malaysia

**Keywords:** Tuberous sclerosis complex, Rapamycin, Rapalogs, Systematic review, Angiomyolipoma, SubEpendymal giant-cell astrocytoma

## Abstract

**Background:**

Rapamycin has gained significant attention for its potential activity in reducing the size of TSC-associated tumors, thus providing alternative to surgery. This study aimed at determining the efficacy of rapamycin and rapalogs for reducing the size of TSC-associated solid tumors in patients with Tuberous Sclerosis Complex (TSC).

**Methods:**

Our data sources included electronic searches of the PubMed. We included into our meta-analysis any type of non-randomized study that reported the use of rapamycin and rapalogs for reducing the size of TSC-associated solid tumors in patients with TSC. Data was entered into Cochrane Review Manager Version 5.3 and analyzed.

**Results:**

Four case reports and 4 clinical trials were included. Five patients from the case reports (all with SEGA) and 91 patients from the clinical trials (41 with SEGA, 63 with kidney angiomyolipoma and 5 with liver angiomyolipoma) were included into the analysis. Volume and diameter of SEGAs were significantly reduced by mean difference of 1.23 cc (95 % CI −2.32 to −0.13; *p* = 0.03) and 7.91 mm (95 % CI −11.82 to −4.01; *p* < 0.0001), respectively. Volume and mean of sum of longest diameter of kidney angiomyolipomas were significantly reduced by mean difference of 39.5 cc (95 % CI −48.85 to −30.15; *p* <0.00001) and 69.03 mm (95 % CI −158.05 to 12.65; *p* = 0.008), respectively. In liver angiomyolipomas, however, reduction in tumor size was not evident. Sum of longest diameter of liver angiomyolipomas in 4 patients were enlarged by 2.7 mm (95 % CI 28.42 to −23.02) by the end of treatment, though not significant (*p* = 0.84).

**Conclusions:**

Rapamycin and rapalogs showed efficacy towards reducing the size of SEGA and kidney angiomyolipoma but not liver angiomyolipomas. This finding is strengthening the conclusion of our Cochrane systematic review on the randomized trials.

## Background

Tumor development is a hallmark in the pathogenesis and diagnosis of Tuberous Sclerosis Complex (TSC). Nine out of 11 major clinical features in TSC diagnosis constitute the appearance of tumor structures [1]. A significant portion of TSC patients showed tumor development. Cortical tubers (CT) occur in 80–90 % [2, 3], subependymal nodules (SEN) in 90 % [4] and subependymal giant cell astrocytoma (SEGA) in 5–20 % of TSC patients. CT has been associated with seizures and developmental delays [5–7], while SEN is normally dormant but may develop into SEGA which potentially causes ventricular obstruction, visual impairment, focal neurologic deficits and endocrinopathies [4, 8].

Kidney angiomyolipoma occurs in 75 % of TSC patients and is a frequent cause of death due to haemorrhage [9]. Another frequent cause of death among TSC patients is pulmonary lymphangioleiomyomatosis (LAM) which occur in 40 % premenopausal females with TSC [10, 9]. Cardiac rhabdomyoma is also commonly found in TSC patients, although generally regress with increasing age [11]. Tumors may also manifest in the skin in the forms of angiofibromas and other cutaneous fibromas [11].

**Fig. 1:**
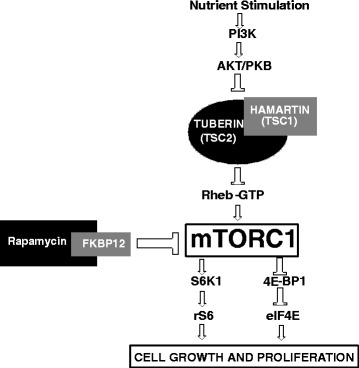
mTOR pathway and inhibition of mTOR through Rapamycin-FKBP12 complex

Development of hamartomatous tumors in TSC patients is related to the mTOR (mammalian target of rapamycin) pathway that acts as a central regulator of many functions, including cell size/growth and proliferation. mTOR combines with several other cellular components to form two distinct complexes, termed mTORC1 and mTORC2 [12], of which only mTORC1 is inhibited by rapamycin and rapalogs. Nutrient stimulation stimulates mTORC1 through activation of PI3 kinase (PI3K) and inhibition of Hamartin/Tuberin Complex. Upregulation of mTORC1 activates S6 kinase1 (S6K1) and ribosomal S6 kinase (rS6), and inhibits eukaryotic translation initiation factor 4E-binding protein 1 (4EBP1) and eukaryotic initiation factor 4E (eIF4E), which results in cell growth and proliferation. (Reviewed in [13]). Figure 1 illustrates the mTOR pathway and mTOR inhibition through rapamycin-FKBP12 complex.

mTOR kinase inhibition was thought to be a useful approach to systemic therapy for TSC and/or LAM because rapamycin, an mTOR inhibitor, has been shown to normalize dysregulated mTOR signaling in cells that lack normal hamartin or tuberin [14–19].

Rapamycin (otherwise known as sirolimus) is an inhibitor of mTOR. In fact, rapamycin sensitivity has been used as the major criterion to identify mTOR-controlled events [20]. Rapamycin (C51H79NO13) is a macrolide compound that was isolated in 1975 from *Streptomyces hygroscopicus* found in a soil sample on Easter Island. It prevents activation of T cells and B cells by inhibiting their response to interleukin-2 (IL-2). It is an FDA-approved drug for immunosuppression after organ transplantation. Rapamycin also possesses both antifungal and antineoplastic properties [21].

The mechanism by which rapamycin inhibits mTOR is not fully understood but rapamycin associates with FKBP12 to bind to the FRB (FKBP12–rapamycin-binding) domain of mTOR. Binding of the rapamycin–FKBP12 complex to mTOR can destabilize the mTORC1 complex and interfere with the activation of mTOR by phosphatidic acid. Several new compounds are available to inhibit mTOR, either by interfering with complex formation (FKBP12-dependent or FKBP12-independent) or by directly inhibiting the catalytic domain of mTOR [22].

A previous study utilizing cohorts of *Tsc2*^*+/−*^mice and mouse model of *Tsc2-null* tumors showed that treatment with an mTOR kinase inhibitor (CCI-779, a rapamycin analogue) reduced the severity of TSC-related disease without significant toxicity [23].

Everolimus, a rapamycin analogue, has been studied in multiple randomized controlled trials for various indications with mostly promising efficacy and safety results such as in de-novo liver transplant patients [24], cardiology patients [25, 26], patients with metastatic renal cell carcinoma [27, 28], patients with neuroendocrine tumors (NET) [29, 30] and breast cancer [31].

A high proportion of tumor manifestations increasingly correspond with the morbidity and mortality due to tumor development in TSC patients. Previous non-human studies have shown the potential application of rapamycin and rapalogs for TSC. Our Cochrane Systematic Review on the randomized studies have shown that there is a significant increase in the proportion of patients who achieved 50 % reduction in tumor size within the subjects group that received rapamycin and rapalogs (Protocol published [32]). However, we have been unable to measure the rapamycin and rapalogs effect on the absolute tumor size, as this latter outcome was only reported in non-randomized studies. Here we analyzed rapamycin and rapalogs effect on the absolute tumor size in patients with Tuberous Sclerosis Complex.

## Methods

There is no published protocol for this systematic review. This systematic review was checked for completeness based on PRISMA 2009 Checklist [33].

### Criteria for considering studies for this review

#### Types of studies

All types of published non-randomized studies (as defined in the Cochrane Handbook version 5.1 [34]) using English language and encountered online through PubMed searches were analyzed.

#### Types of participants

People with known TSC-associated SEGA, kidney angiomyolipoma and/or liver angiomyolipoma as proven by the clinical features designated in the 2012 consensus diagnostic criteria for TSC and/or TSC-causing mutations in either TSC1 or TSC2 gene [1]. Studies and/or participants without solid tumors or non-TSC-associated tumors were excluded.

#### Types of interventions

Any rapamycin or its analogues (rapalogs) designed to reduce the size of TSC-associated tumors in patients with Tuberous Sclerosis Complex

#### Types of outcome measures

We chose tumor volume or diameter as primary outcome. We also reported adverse effects whenever they are described as related to the rapamycin and rapalogs administration.

### Search methods for identification of studies

Electronic searches in PubMed used keywords “TSC AND [SEGA OR kidney angiomyolipoma OR liver angiomyolipoma] AND [rapamycin OR sirolimus OR tacrolimus OR everolimus]”. All published articles and abstracts were searched. The search was limited to reports on human studies using English language.

### Data collection and analysis

#### Statistical analysis

Available data (mean and standard deviation of each cohort study and pooled case reports) was entered into Cochrane Review Manager Version 5.3 [35] for analysis of treatment effects.

#### Selection of studies

**Fig. 2:**
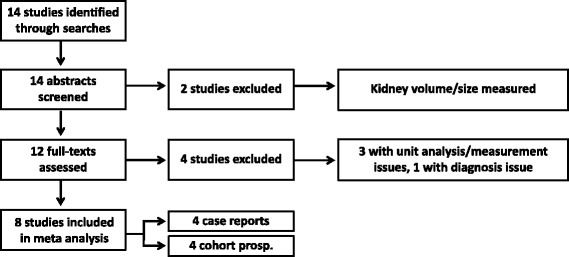
Study flow diagram (based on The PRISMA Statement [33])

Studies were selected according to the criteria for considering studies for this review, as described above. Please refer to the PRISMA diagram illustrating the study selection (Fig. 2).

#### Data extraction

Data was extracted using the specially designed data acquisition form as used in the Cystic Fibrosis and Genetic Disorders Group of The Cochrane Collaboration. The following items were extracted : type of study, participants and trial characteristics (single/multi-center, countries, eligibility, number of participants in the study, number of participants included in this review), intervention details (type of rapamycin or rapalogs, administering dose, trough level, duration of treatment, median or range of follow-up), outcomes (tumor volume and/or diameter) and rapamycin-related adverse effects.

#### Risk of bias assessment

The risk of bias of each included study was assessed using the 8-item Newcastle-Ottawa Scale for observational studies [36].

#### Measures of treatment effect

We recorded continuous data such as tumor volumes or diameter as mean baseline and post treatment values and standard deviation (SD) for each group. We calculated a pooled estimate of the treatment effect at the end of each core length of treatment for each outcome across studies by determining the mean difference. We pooled outcome data from case reports into one group.

#### Assessment of heterogeneity

Test for heterogeneity between studies was done using a standard Chi^2^ test and I^2^ statistic [37]. The Chi^2^ test is a statistical test for heterogeneity, whereas I^2^ assesses the quantity of inconsistency across studies in the meta-analysis. We used a cut-off *P* value of 0.1 to determine significance. This is because of generally anticipated low power of the reported trials due to the disease being rare. We used the following I^2^ ranges to interpret heterogeneity: 0 to 40 %: might not be important, 30 to 60 %: may represent moderate heterogeneity, 50 to 90 %: may represent substantial heterogeneity and 75 to 100 %: considerable heterogeneity.

## Results and discussion

### Characteristic of studies

#### Results of the search

Fourteen studies were identified from the searches (Fig. 2). Screening of abstracts excluded two studies [38, 39] because the studies measured the organ’s volume/size instead of the tumor’s. These are case reports with one patient each. Further full-text assessments excluded four studies with unit analysis/measurement or diagnosis issues [40–43]. One was a prospective cohort study involving 25 patients with angiomyolipoma or lymphangioleiomyomatosis where the TSC-diagnosed patients were mixed up with non TSC-diagnosed patients [40] and the rest were case reports with 1 patient each [41–43]. This review finally included eight studies consisting of four case reports [44–47] and four prospective cohort studies [48–51].

#### Included studies (Table 1)

**Table 1:** Characteristics of included studies

Study type		Case report				Cohort prospective			
Studies		Franz 2006	Wienecke 2006	Birca 2010	Lam 2010	Krueger 2010	Dabora 2011	Lopez 2011	Davies 2011
n^a^		3	1	1	1	28	36	17	10
Sex	M	1	1		1	17	10	8	NS
	F	2		1		11	26	9	
Age range (years)		14.5 – 21	19	8	9	3 – 34	19 – 60	>10	NS
Genotype	TSC1					4			
	TSC2	3	1	1	1	10	14		
	Unknown					14	22	17	10
Rapamycin	Dose (mg/day)	2 – 7	0.5	1	5 – 7	3^b^	2 – 6	1	0.5^c^
	Trough (ng/ml)	7.7 – 10.9	4 – 5	3.3 – 4.5	10 – 15	5 – 15	3 – 15	4 – 8^d^	3 – 6

*NS* Not specified

^a^Number of patients included in the analyses

^b^mg/m^2^

^c^mg/m^2^/day

^d^ng/dl

### Characteristics of case reports

Franz et al. [44] reported four patients with SEGA altogether. However, only three patients (1 male 2 females, aged 14.5 – 21 years old) were reported with mutations in TSC2 gene. We excluded one patient of this report due to unclear TSC diagnostic data. The patients were treated with rapamycin 2 – 6 mg/day to achieve trough level of 7.7 – 10.9 ng/ml for 2.5 – 20 months.

Wienecke et al. [47] reported 1 patient (male, aged 19 years old) with kidney angiomyolipoma, in whom a mutation of TSC2 was identified. The patient was treated with rapamycin 1 mg every other day – 2 mg/day to reach trough level of 4 – 5 ng/ml for 7 months. Birca et al. [45] reported 1 patient (female, 8 years old) with SEGA. The patient showed clinical features of TSC as well as continuous deletion of TSC2/PKD1 region. Treatment with rapamycin was 1 – 2 mg/day to reach trough level of 3.3 – 4.5 ng/ml. Treatment duration was not mentioned, but last follow-up was after 5 months of treatment. Lam et al. [46] reported 3 patients with TSC-associated SEGA and kidney angiomyolipoma. However, we could only analyzed 1 patient (male, 9 years old) since the remaining 2 patients were reported as percentage decrease in tumor size and not the absolute value of tumor volume or diameter. The patient was treated with rapamycin 5 – 7 mg/day (in combination with carbamazepine that is known to induce rapamycin metabolism) to reach trough level of 10 – 15 ng/ml for a year. However, the follow-up reported was only that of 3 months.

### Characteristics of prospective cohort studies

Krueger et al. [51] was described as a prospective, open-label, phase I-II trial. It was carried out in a single center at the Cincinnati Children’s Hospital Medical Center (US). The study reported 28 patients (17 males and 11 females; aged 3 – 34 years old) with definitive diagnosis of TSC and SEGA. The patients were treated with Everolimus orally at a starting daily dose of 3.0 mg per square meter of body-surface area and were subsequently adjusted to attain a whole-blood trough concentration of 5 – 15 ng/ml. The participants were essentially treated for 6 months with a median length of follow-up of three months.

Dabora et al. [49] was described as an open label, single arm, multicenter study with a Simon two-stage design [52]. It was carried out in six clinical centers at the United States (Boston, Cincinnati, Loma Linda, Hartford, New York and Dallas). The study reported 36 patients (10 males and 26 females; aged 19 – 60 years old) with definitive diagnosis of TSC, all with kidney angiomyolipoma, 13 with SEGA and 4 with liver angiomyolipoma. The patients were treated with Sirolimus orally at a loading dose of 6 mg followed by 2 mg/day to achieve trough level of 3 – 9 ng/ml during the first 16 weeks. After 16 weeks, the dose was increased to achieve a trough level of 9 – 15 ng/ml. The treatment lasted for 12 months with a median length of follow-up of 4 months.

Cabrera-Lopez et al. [48] was described as a phase IV non-blinded, non-controlled clinical trial. It was carried out at a single center in Spain. The study reported 17 patients (8 males and 9 females older than 10 years of age) with TSC diagnosis showing kidney angiomyolipoma. The patients were treated with rapamycin with initial dose of 1 mg/day. The dosage was increased 1 mg/day every for two weeks until it reached stable trough concentration of 4 – 8 ng/dl. The treatment lasted for 12 months with a median length of follow-up of 6 months.

Davies et al. [50] was described as phase II non-randomized, open-label multicenter trial. It was carried out in 4 hospitals of the United Kingdom and Switzerland. The study reported 16 patients, out of which only 10 patients were with definitive diagnosis of TSC and showing kidney angiomyolipoma. The patients were treated orally with sirolimus at initial dosage of 0.5 mg/square meter of body surface. The dosage was adjusted to achieve trough concentration of 3 – 6 ng/ml. The treatment lasted for 24 months with a median length of follow-up of 5 months.

#### Risk of bias assessment

The results of the risk of bias assessments for individual studies are reported in Table 2. Overall, each study showed high to low risk of bias.

**Table 2:** Risk of bias assessment of included studies

Studies	Representativeness of exposed cohort	Selection of non-exposed cohort	Ascertainment of exposure	Demonstration	Comparability	Assessment of outcome	Follow-up long enough^a^	Adequacy of follow-up^b^	Total score
Case Reports									
Franz 2006	Somewhat representative^*^	No non-exposed cohort	Secure record^*^	Yes^*^	No	Record linkage^*^	Variable	Complete^*^	5
Wienecke 2006	Somewhat representative^*^	No non-exposed cohort	Secure record^*^	Yes^*^	No	Record linkage^*^	Yes^*^	Complete^*^	6
Birca 2010	Somewhat representative^*^	No non-exposed cohort	Secure record^*^	Yes^*^	No	Record linkage^*^	Unclear	Complete^*^	5
Lam 2010	Somewhat representative^*^	No non-exposed cohort	Secure record^*^	Yes^*^	No	Record linkage^*^	Yes^*^	Complete^*^	6
Cohort Prospective									
Krueger 2010	Truly representative^*^	No non-exposed cohort	Secure record^*^	Yes^*^	No	Record linkage^*^	Yes^*^	Complete^*^	6
Dabora 2011	Truly representative^*^	No non-exposed cohort	Secure record^*^	Yes^*^	No	Record linkage^*^	Yes^*^	8 out of 36 lost follow-up (22 %)^*^	6
Davies 2011	Truly representative^*^	No non-exposed cohort	Secure record^*^	Yes^*^	No	Record linkage^*^	Yes^*^	4 out of 16 lost follow-up (25 %)^*^	6
Lopez 2011	Truly representative^*^	No non-exposed cohort	Secure record^*^	Yes^*^	No	Record linkage^*^	Yes^*^	1 out of 17 lost follow-up (5 %)^*^	6

^a^Length of follow-up of at least 6 months is considered sufficient

^b^Lost of follow-up <30 % is considered acceptable; *Indicates point that contribute to Total Score

### Effects of interventions on tumor size

Five patients from the case reports (all with SEGA) and 91 patients from the clinical trials (41 with SEGA, 63 with kidney angiomyolipoma and 5 with liver angiomyolipoma) were included into the analysis. An additional 3 patients from the case reports (1 with kidney angiomyolipoma [47] and 2 with SEGA [46]) were described, albeit not systematically analyzed.

#### SubEpendymal Giant cell Astrocytoma (SEGA) (Table 3)

In total, there are 5 studies that looked at the effect of rapamycin or rapalogs on SEGA, in which 3 are case reports [44–46] and 2 are prospective cohort [49, 51]. Two studies used tumor volume (cc) [51, 44] and 3 studies used tumor diameter (mm) [49, 45, 46] as their unit of analysis.

**Table 3:** Outcome on Subependymal Giant Cell Astrocytoma (SEGA)

Studies	Volume (cc)					Diameter (mm)				
	Baseline	n	After treatment	n	Mean diff (95 % CI)	Baseline	n	After treatment	n	Mean diff (95 % CI)
Case Reports	2.9 ± 2.4	3	1.2 ± 0.9	3	−1.7 (−4.6, 1.2)	35 ± 3.7	2	23 ± 1.8	2	−12.00 (−17.70, −6.30)
Kruger 2010	2.45 ± 2.81	28	1.3 ± 1.48	27	−1.01 (−2.33, 0.03)					
Dabora 2011						16.7 ± 6.4	13	12.4 ± 6.9	11	−4.3 (−9.66, 1.06)
Overall Effect					−1.23 (−2.32, −0.13)					−7.91 (−11.82, −4.01)
					*p* = 0.03					p < 0.0001
Heterogeneity					Chi^2^ = 0.12; I^2^ = 0 %					Chi^2^ = 3.72; I^2^ = 73 %

The case reports [44–46] comprised of 5 patients with SEGA. From Franz et al. [44] we included 3 patients using tumor volume (cc) as unit of analysis. Lengths of treatment are variable, ranging from 2.5 – 20 months. Reduction in the tumor volume was noted in all patients, with a mean difference of −1.7 cc (95 % CI −4.6 to 1.2; *p* = 0.3). Birca et al. [45] reported one patient on which we took the longest tumor diameter (mm) as unit of analysis. Length of treatment was 5 months. There are two SEGAs found in this patient, from which we took the average for analysis. At 3-month follow-up tumor 1 was reduced from 38.7 mm to 24.3 mm while tumor 2 was also reduced from 31.3 mm to 21.8 mm. No further reduction in tumor size was noted at 5-month follow-up. From Lam et al. [46] we analyzed only one out of 3 patients using tumor longest diameter (mm) as unit of analysis. Length of treatment was 9 months. At 3-month follow-up the tumor was reduced from 35 mm – 26 mm. No further reduction in tumor size was noted at 6- and 9-month follow-ups. Reduction in the tumor diameter was noted in these 2 patients, with a mean difference of −12.00 mm (95 % CI −17.70 to −6.30; *p* = 0.05). The other 2 patients showed significant reduction in tumor size of 50–60 % of the pre-treatment size [46].

Dabora et al. [49] reported 13 patients with measurable SEGA ranging in diameter from 9–30 mm. However, follow-up data was only available from 11 patients after 12 months of Sirolimus treatment. Tumor regression was observed in 7 cases and SEGA diameter was stable in the other 4 cases. Our analysis showed that the SEGA in these 11 patients were reduced by mean difference of −4.3 mm (95 % CI −9.66 to 1.06; *p* = 0.1).

Krueger et al. [51] reported 28 patients with SEGA whereby one patient was withdrawn at 4.5 months. This study reported two versions of assessment at 6-month of follow-up; that of the local investigator’s and that of the independent central review. We include only the latter in our analysis. Local investigator’s assessment examined the tumor volume (cc) at 3, 6, 12, 18 and 24 months. We noted fluctuation in the mean of tumor volume reduction of 3 months (0.88 cc), 6 months (1.04 cc), 12 months (0.99 cc), 18 months (1.11 cc) and 24 months (0.87 cc) as compared to baseline. Our analysis showed that SEGA in 27 patients after 6 months of follow-up were reduced by mean difference of −1.01 (95 % CI −2.33 to 0.03; *p* = 0.06).

Overall, volume of SEGAs in 30 patients was significantly reduced (*p* = 0.03) after 3–12 months therapy, by mean difference of −1.23 cc (95 % CI −2.32 to −0.13). Similar pattern was also noted in the diameter of SEGAs in 13 patients (*p* < 0.0001) after 5–12 months treatment, by mean difference of −7.91 mm (95 % CI −11.82 to −4.01).

#### Kidney angiomyolipoma (Table 4)

There are 4 studies that looked at the effect of rapamycin on kidney angiomyolipoma. One is a case report [47] and three are cohort prospective [48–50]. Cabrera Lopez et al. [48] and Wienecke et al. [47] used tumor volume (cc), while Dabora et al. [49] and Davies et al. [50] used tumor diameter (mm) as their unit of analysis.

**Table 4:** Outcome on kidney angiomyolipoma

Studies	Volume (cc)					Sum of longest diameter (mm)				
	Baseline	n	After treatment	n	Mean diff (95 % CI)	Baseline	n	After treatment	n	Mean diff (95 % CI)
Cabrera Lopez 2011	62.6 ± 18.3	17	23.1 ± 7.0	16	−39.5 (−30.15,–48.85)					
Dabora 2011						212 ± 146	36	145 ± 113	28	−67 (−3.55,–130.5)
Davies 2011						135.5 ± 135.6	10	62.8 ± 20.1	7	−69.80 (15.39,–154.99)
Overall Effect					−39.5 (−48.85, −30.15)					−69.03 (−158.05, 12.65)
					*p* = <0.00001					*p* = 0.008
Heterogeneity					Not applicable					Chi^2^ = 0.01; I^2^ = 0 %

Wienecke et al. [47] reported 1 patient with baseline tumor volume of 134 cc before administration of Rapamycin. Five months following Rapamycin therapy, MRI showed the tumor shrunk to 39 cc. The tumor volume was further reduced to 23 cc at 7 months following treatment. At 7 months, Rapamycin treatment was terminated. As a result, 15 months after the initial treatment, the tumor re-grew to 116 cc, following which rapamycin was re-instutited. A rapid decrease in tumor volume (to 45 cc) was noted after 3 months.

Cabrera Lopez et al. [48] reported 17 TSC patients with kidney angiomyolipoma, out of whom one had to be withdrawn from the study after 11 months due to reactivation of an erythema nodosum which was already present at the start of the trial, and another patient was removed from the study after 13 months of treatment due to the appearance of nephrotic proteinuria which disappeared after treatment suspension. Length of treatment was 24 months. Tumor volume was examined at 6-month and 12-month following the treatment. At 6-month follow-up the tumor size was significantly decreased by mean difference −34.00 cc (95 % CI 24.62 to −43.38; *p* = 0.0000). The tumor size further shrunk at 12-month follow up by mean difference of −39.50 cc (95 % CI −48.85 to −30.15; *p* = <0.00001).

Dabora et al. [49] reported 36 TSC patients with kidney angiomyolipoma, out of whom 8 were withdrawn from the study. These included 4 who were unable to come for study appointments, and 4 who were taken off study because of a serious adverse event that was judged unrelated to drug therapy (1 hospitalized with complicated infectious mononucleosis, 1 diagnosed with a pancreatic neuroendocrine tumor, 1 hospitalized with a kidney angiomyolipoma hemorrhage, 1 hospitalized with a neurologic event). Core length of treatment was 12 months. Follow-ups were done at 4, 8 and 12 months. Sum of the longest diameter (SLD) of the tumor (mm) was used as unit of analysis. At 4-month follow up (n = 33), the tumor SLD decreased but not significantly (*p* = 0.28) by mean difference of −36.00 mm (95 % CI 28.67 to −100.67). At 8-month follow-up (n = 29), the tumor SLD further decreased (*p* = 0.05) by mean difference of −62.00 mm (95 % CI 0.04 to −124.04). At 12-month follow-up (n = 28), the tumor SLD further decreased (*p* = 0.05) by mean difference of −67.00 mm (95 % CI −3.55 to −130.45).

Davies et al. [50] reported 10 TSC patients with kidney angiomyolipoma, out of whom 3 were withdrawn. Length of treatment was 24 months. SLD of the tumors was taken as unit of analysis. Follow-ups were done at 2,6,12 and 24 months. At 2 months (n = 9), the tumor SLD was decreased (*p* = 0.9) by mean difference of −8.00 mm (95 % CI 118.15 to −134.15). At 6 months (n = 10), the tumor SLD was further decreased (*p* = 0.79) by mean difference of −16.3 mm (95 % CI 103.13 to −135.73). At 12 months (n = 8), the tumor SLD was decreased (*p* = 0.11) by mean difference of −69.8 mm (95 % CI 15.39 to −154.99). Again, at 24 months (n = 7), the tumor was decreased further (*p* = 0.2) by mean difference of −69.8 mm (95 % CI 15.39 to −154.99). It is of note that at follow-ups of 12 and 24 months, 3 patients with 18 tumor lesions were withdrawn.

Overall, volume of kidney angiomyolipoma in 16 patients was significantly reduced (*p* <0.00001) by mean difference of −39.5 cc (95 % CI −48.85 to −30.15) after 12 months rapamycin therapy. This reduction was also noted in the mean of SLD of the kidney angiomyolipomas in 35 patients (*p* = 0.008) by −69.03 mm (95 % CI −158.05 to 12.65). In total, 11 patients with kidney angiomyolipomas were withdrawn before 12 months of therapy.

#### Liver angiomyolipoma

There is only one study that looked into the effect of rapamycin on liver angiomyolipoma, which is a prospective cohort [49]. This study used the tumor’s SLD as the analysis unit. There are 5 TSC patients with measurable liver angiomyolipoma, out of which 1 was withdrawn before 12 months of treatment. Core length of treatment was 12 months. Follow-ups were done at 4, 8 and 12 months. At 4-month follow-up (n = 4), the tumor SLD was decreased (*p* = 0.05) by mean difference of −12.80 mm (95 % CI −0.1 to −25.5). At 8-month follow-up (n = 4), the tumor SLD was still smaller as compared to that of baseline (*p* = 0.08) by mean difference of −12.5 mm (95 % CI 1.33 to −26.33) but this slightly re-grew as compared to that at 4-month follow-up. At 12-month follow-up, the tumor SLD was slightly larger (*p* = 0.84) as compared to that of baseline by mean difference of 2.7 mm (95 % CI 28.42 to −23.02). Reduction of tumor size in liver angiomyolipoma was not evident.

### Adverse effects

Summary of adverse effects found in 99 patients from the included studies was described in Table 5. The most frequent adverse effects were oral ulcer followed by hypertriglyceridemia and upper respiratory tract infection.

**Table 5:** Adverse effects in 99 patients

Adverse effect	Percentage of patients
Oral ulcer	54.5
Hypertriglyceridemia	28.3
Upper Respiratory Tract Infection	26.3
Acne/Acneiform Dermatitis	19.2
Sinusitis	16.2
Proteinuria	16.2
Hypercholesterolemia	15.2
Tinea/Other Skin problem	14.1
Leucocytosis	14.1
Diarrhoea	12.1
Anemia	12.1
Otitis Media	10.1
Pyrexia	8.1
Cellulitis	8.1
Joint pain	8.1
Urinary Tract Infection	7.1
Headache	6.1
Lymphopenia	6.1
Increased Alkaline Phosphatase	5.1
Irregular menses	5.1
Nose bleeding	5.1
Gastroenteritis	4
Otitis Externa	4
Incerased ALT, SGPT	4
Gastric Infection	3
Cough	3
Increased Creatinine Kinase	3
Peripheral Oedema	3
Fatigue	3
Vomiting	2
Gingival Hypertrophy	1
Myalgia	1
Acute Pyelonehpritis	1
Dry skin	1
Depression	1
Weight gain	1
Hypothyroidism	1
Retinal tear	1

We identified, in this systematic review, 8 studies that fulfill our criteria for study inclusion involving a total of 99 trial participants. Six patients from the case reports (5 with SEGA, 1 with kidney angiomyolipoma) and 91 patients from the prospective cohorts (41 with SEGA, 63 with kidney angiomyolipoma and 5 with liver angiomyolipoma) were included into the analysis. Two other patients were described from a case report, albeit not analyzed.

On the quality of the included studies, we admit that we only included in this analysis non-randomized studies which by itself were a limitation. However, although the case reports may be of moderate to high risk of bias, the prospective cohort studies that we included may be categorized into moderate to low risk of bias, to the extent possible as non-randomized studies. There is a notable variability in the duration of follow-up, which might affect treatment outcome.

Thirty-one patients with SEGA were analyzed for their tumor volume (cc), while 15 patients for their tumor diameter. Analyses using both units showed a significant reduction in the size of SEGAs following the treatment. The volume was significantly reduced after 3 – 12 months of rapamycin administration by 1.23 cc and 2.45 stretch of reduction interval. The diameter, on the other hand, showed stretch of reduction interval of 7.81 and a mean reduction of 7.91 mm after 5 – 12 months therapy.

Forty-six patients with kidney angiomyolipomas were analyzed of their tumor’s sum of longest diameter (mm), while 17 patients of their tumor volume (cc). Both analyses showed significant reduction in the size of kidney angiomyolipoma following the treatment. The sum of longest diameters was significantly reduced by 69.03 mm and a stretch of reduction interval of 170.7. The volume was also significantly reduced by 39.50 cc with a stretch of reduction interval of 18.7. However, there are 12 patients withdrawn from the study before completion of 12 months therapy.

These have shown that rapamycin and rapalogs significantly reduce the tumor size in Tuberous Sclerosis patients with SEGA and kidney angiomyolipomas. However, the same reduction in tumor size was not evident in liver angiomyolipomas which involve only a limited set of patients.

Rapamycin has also been tried on other manifestations of Tuberous Sclerosis Complex with mostly promising results, albeit variable degree of evidences. Reports described improvement in facial angiofibroma [53–56], epilepsy [57, 58], cardiac rhabdomyoma [59], lymphangioleiomyomatosis [40, 50], fibromatosis and renal cell carcinoma [60] but not the optic nerve tumor [42] among patients with Tuberous Sclerosis Complex.

Our Cochrane systematic review (Protocol published [32]) on the same treatment effect which included only randomized studies [61–63] showed that there is a significant increase in the proportion of patients who achieved 50 % reduction in tumor size within the participants group that received rapamycin and rapalogs. This used dichotomous data for its analyses. The current article meta-analyzed non-randomized studies for the effect of rapamycin and rapalogs on tumor size and used continuous data for its analyses (actual tumor volume and diameter).

Adverse effects of the rapamycin treatment should be highlighted given that slightly more than half of the patients showed at least one of the listed adverse effects (Table 5). However, only very few were reported to develop serious conditions [49, 51].

## Conclusion

As a conclusion, we have shown that rapamycin and rapalogs reduce the size of SEGA and kidney angiomyolipoma in patients with Tuberous Sclerosis Complex. This result strengthens our Cochrane systematic review on randomized studies. However, it is of note that we are unable to rule out the possibility of publication bias where authors only report positive outcomes and there may be many instances where no response was unreported.
